# Evaluating Diuretics in Normal Care (EVIDENCE): protocol of a cluster randomised controlled equivalence trial of prescribing policy to compare the effectiveness of thiazide-type diuretics in hypertension

**DOI:** 10.1186/s13063-021-05782-9

**Published:** 2021-11-17

**Authors:** Amy Rogers, Angela Flynn, Isla S. Mackenzie, Lewis McConnachie, Rebecca Barr, Robert W. V. Flynn, Steve Morant, Thomas M. MacDonald, Alexander Doney

**Affiliations:** MEMO Research, University of Dundee, Ninewells Hospital and Medical School, Dundee, DD1 9SY UK

**Keywords:** Medical record linkage, Comparative effectiveness research, Drug prescriptions, Primary health care, Hypertension

## Abstract

**Introduction:**

Healthcare systems must use treatments that are effective and safe. Regulators licensed many currently used older medications before introducing the stringent evidential requirements imposed on modern treatments. Also, there has been little encouragement to carry out within-class, head-to-head comparisons of licensed medicines. For commonly prescribed drugs, even small differences in effectiveness or safety could have significant public health implications. However, conventional clinical trials that randomise individual subjects are costly and unwieldy. Such trials are also often criticised as having low external validity. We describe an approach to rapidly generate externally valid evidence of comparative safety and effectiveness using the example of two widely used diuretics for the management of hypertension.

**Methods and analysis:**

The EVIDENCE (Evaluating Diuretics in Normal Care) study has a prospective, cluster-randomised, open-label, blinded end-point design. By randomising prescribing policy in primary care practices, the study compares the safety and effectiveness of commonly used diuretics in treating hypertension. Participating practices are randomised 1:1 to a policy of prescribing either indapamide or bendroflumethiazide when clinically indicated. Suitable patients who are not already taking the policy diuretic are switched accordingly. All patients taking the study medications are written to explaining the rationale for changing the prescribing policy and notifying them they can opt-out of any switch. The prescribing policies’ effectiveness and safety will be compared using rates of major adverse cardiovascular events (hospitalisation with myocardial infarction, heart failure or stroke or cardiovascular death), routinely collected in national healthcare administrative datasets. The study will seek to recruit 250 practices to provide a study population of approximately 50,000 individuals with a mean follow-up time of two years. A primary intention-to-treat time-to-event analysis will be used to estimate the relative effect of the two policies.

**Ethics and dissemination:**

EVIDENCE has been approved by the East of Scotland Research Ethics Service (17/ES/0016, current approved protocol version 5, 26 August 2021). The results will be disseminated widely in peer reviewed journals, guideline committees, National Health Service (NHS) organisations and patient groups.

**Trial registration:**

ISRCTN46635087. Registered on 11 August 2017 (pre-recruitment).

## Background

Formal comparisons of the effectiveness and safety of medicines with similar modes of action and indication are rare [1]. As the number of available medicines increases, high-quality evidence of comparative effectiveness becomes increasingly essential. This problem was the subject of two recent Lancet reviews and accompanying commentary that promoted comparative effectiveness research for public health [2–4].

Randomised placebo-controlled trials (RCTs), often considered the gold standard for generating healthcare evidence, can be cumbersome, expensive and time-consuming [5]. Such trials often have low external validity because trial participants are likely to differ from patients encountered in usual healthcare practice [6]. As it is conventionally conducted, the randomised controlled trial is not a suitable tool for generating comparative effectiveness evidence at the required scale and speed. A more efficient method that can generate evidence within acceptable boundaries of precision and within reasonable time frames and resources is needed.

The potential for using routinely collected data to generate knowledge within a learning healthcare framework is increasingly recognised [7]. Whilst such data can be used to produce evidence quickly and efficiently, non-interventional research is subject to biases that limit its usefulness for clinical decision making [8]. The result is, despite significant advances in causal inference techniques [9, 10], purely observational research often fails to influence policy and behaviour [8]. Researchers can enhance the value of these large datasets by combining them with the essential features of randomised trials and healthcare system-specific processes.

We describe a study design that uses routinely collected healthcare data in combination with cluster randomisation to generate high-quality evidence of comparative effectiveness of prescribing policy efficiently and rapidly. Using the example of thiazide-type diuretic medications, widely used for managing hypertension, the EVIDENCE (EValuatIng DiurEtics in Normal CarE) study will compare policies for prescribing indapamide or bendroflumethiazide. Previous work by our group has demonstrated good public support for this type of research [11]. Whilst we have developed the EVIDENCE protocol using the exemplar of diuretic medicines, the infrastructure and methodology will be applicable to the wide range of situations where comparative safety and effectiveness evidence is lacking for treatments commonly used in the NHS.

### Rationale for EVIDENCE

Cardiovascular diseases are the leading cause of death worldwide with high blood pressure being the most common preventable cause, responsible for 54% of strokes and 47% of ischaemic heart disease [12]. Given the high prevalence of hypertension, affecting around 12.5 million people in England in 2015 [13], even slight differences in the effectiveness or safety between widely prescribed blood pressure-lowering medications would imply many thousands of potentially avoidable events.

Thiazide and thiazide-like diuretics are a cornerstone of hypertension treatment. The scant evidence for clinically relevant differences between the thiazide and thiazide-like diuretics has been interpreted variably by blood pressure management guidelines internationally [14]. In 2011, the UK NICE guidelines for managing hypertension [15] stated that “a thiazide-like diuretic, chlortalidone or indapamide, should be chosen in preference to a conventional thiazide diuretic such as bendroflumethiazide or hydrochlorothiazide”. The guidelines development group (GDG) conceded that “there were no direct comparisons between the different diuretics with regard to clinical outcomes” and that “the GDG found it difficult to reach firm conclusions regarding the comparative efficacy of different thiazide-type diuretics with regard to blood pressure-lowering” [15]. Before this guidance, UK diuretic use for hypertension was almost entirely restricted to bendroflumethiazide [16, 17]. The 2011 guideline was therefore suggesting a significant change to the prevalent prescribing of these drugs. The 2019 guideline update, NG136, still recommends a thiazide-like diuretic in preference to a conventional thiazide [18].

The interpretation of the evidence by the GDG has been disputed [19]. A recent meta-analysis from our group has further demonstrated the lack of evidence in this area [20]. Despite NICE guidance, bendroflumethiazide remains the dominant diuretic prescribed in the UK to treat hypertension [16]. The hypertension community remains in equipoise about diuretic choice, and many local prescribing policies did not take up the NICE recommendation. This situation is likely to have also been influenced by chlortalidone not being readily available in the UK and indapamide being significantly more expensive than bendroflumethiazide [21].

To conduct a conventional individually randomised clinical trial comparing the effects of bendroflumethiazide and indapamide on cardiovascular outcomes would be very challenging; such a trial would likely require the recruitment of over 50,000 individual patients, making it one of the largest hypertension trials ever. Furthermore, as both drugs are now off-patent, securing sufficient funding to achieve this level of recruitment for this comparison and many others within current health research funding structures would be a formidable task. Therefore, we propose the EVIDENCE trial design as a pragmatic solution to the problem of how to conduct comparative effectiveness research within existing constraints.

### Objectives

The primary objective of EVIDENCE is to estimate the relative effectiveness of two prescribing policies for the management of hypertension in terms of both safety and efficacy. If study results suggest that there is no clinically relevant difference [22], this will justify individual prescribers and healthcare providers continuing to decide which drug to offer based on other relevant factors such as availability and price.

Our secondary objective is to evaluate the feasibility of the EVIDENCE method for conducting clinical effectiveness research in the NHS. We intend that this methodology be adapted to many disease areas as part of a future learning NHS.

## Methods and analysis

### Study design

EVIDENCE is a cluster-randomised, prospective, parallel-group, blinded outcome study comparing cardiovascular event rates in patients in general practices being prescribed either bendroflumethiazide or indapamide to manage hypertension.

The cluster randomised approach has been chosen to increase the feasibility of performing comparative effectiveness research at scale in the NHS; the cost of conducting multiple comparisons of commonly used medicines using individual patient recruitment and randomisation would be prohibitive. However, this feasibility advantage must be balanced against a need for careful statistical planning [23, 24].

There is, nonetheless, an important advantage of cluster randomisation without individual opt-in consent over more traditional approaches: using an opt-out of intervention approach is likely to result in a study cohort far more representative of the intended patient population [25, 26].

**Fig. 1:**
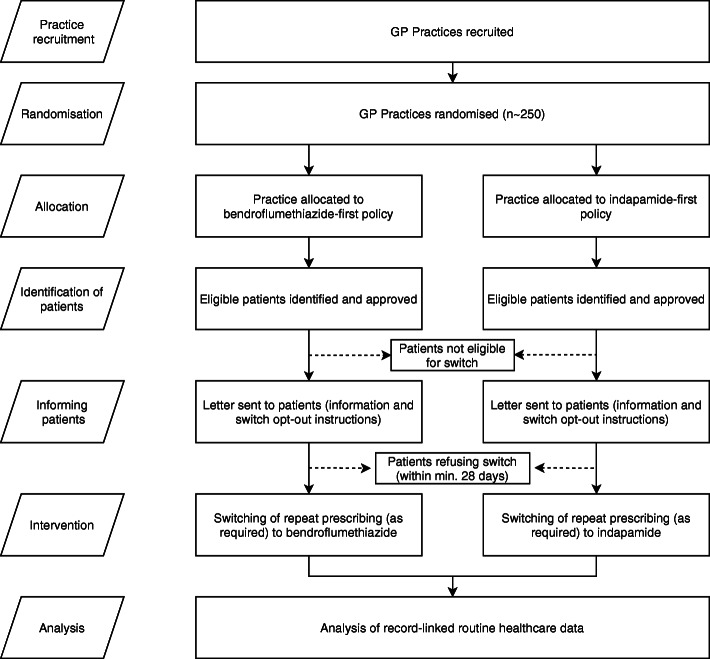
EVIDENCE study flow diagram

NHS general medical practices using electronic medical records will be invited to take part in the study. The study will recruit practices from a broad range of NHS trusts, including remote and rural practices that are often excluded from traditional trial participation. The study is designed to be minimally disruptive to practice workflow and uses routine prescribing activities. Recruitment of general practices and randomisation of prescribing policy at the practice level allows the rapid accrual of a large observational study population whilst incorporating the statistical benefits of randomisation. Routinely collected national datasets of hospitalisations and deaths will be used to compare cardiovascular event rates between individuals being treated under each of the two policies. National dispensed prescribing information will be used to facilitate the definition of analysis cohorts. General practice data will be used to gather evidence on potentially important adverse effects such as electrolyte and metabolic disturbances. The study will take place in Scotland where national datasets are well established, although we anticipate that extension to other regions of the UK would be feasible. Figure 1 shows the EVIDENCE study flow diagram.

### Identification of the study population

A search will be performed in each recruited practice using a bespoke EHR-specific tool to identify patients who may be subject to a medication switch after policy randomisation based on the following criteria.

#### Inclusion criteria

- Documented diagnosis of hypertension (on practice hypertension register)
- Currently receiving repeat prescriptions for bendroflumethiazide or indapamide
- Aged over 18

#### Exclusion criteria

- Documented history of an adverse drug reaction to either medication
- History of having been prescribed both thiazide-like diuretics at different times, indicating a potential clinical reason for not being able to take one or other
- Other cogent clinical or other indication for not switching medication (see below)

A draft list of potential patients identified in each practice will be provided to a relevant member of the primary care team (a practice GP or delegate) for approval. Approval will include the redaction of any individuals considered not suitable for the drug switch, based on local knowledge. The approved list will be used for the study and represents the study population for the practice.

### Cluster randomisation

The randomisation unit in this study is the practice (the cluster); patients are not individually recruited. Following identification and approval of the practice study population, the practice will be randomised using an online study portal. Randomisation will be 1:1, block balanced by practice list size, using a computerised randomisation algorithm. Block sizes are randomly allocated to prevent staff implementing the trial from guessing the next allocation. The prescribing policies will be applied at a practice level, and individual prescribers will remain free to prescribe as clinically appropriate.

## Intervention

### Policy medications

- Bendroflumethiazide is licensed for the treatment of hypertension and oedema, although by far the most common indication is hypertension. The typical dose for hypertension is 2.5 mg once daily. A small minority of patients are prescribed 5 mg or 1.25 mg daily.
- Indapamide is licensed to treat hypertension only at 2.5 mg/day, although tablets are scored and 1.25 mg can be taken. It is also available as a more expensive slow-release preparation at a dose of 1.5 mg. The 2.5 mg standard release formulation will be used in this study.

### Policy implementation

#### Informing patients about the study

Immediately following randomisation of a practice, all patients in the study population will be written to informing them that their practice is taking part in the EVIDENCE study. The letter will be printed on practice-specific headed paper and will briefly explain why the study is being conducted. It will also advise that the patient's repeat prescriptions for thiazide-type medication may be switched in line with the newly assigned practice prescribing policy. The letter directs patients who may have any questions, concerns, or objections to visit a study-specific website (www.memoresearch.com/evidence) or directly contact the study team. The study team will provide further information and, if the patient confirms that they would prefer to opt-out of any switch and remain on their current medication, the study team will not action the switch for that individual.

#### Repeat prescription switching

Patients whose current medication prescription is concordant with the randomly assigned policy will remain on their existing strength and dosage of thiazide-type diuretic. Where a patient is not being prescribed for in concordance with the randomly assigned policy, future repeat prescriptions will be altered as follows:

1. Bendroflumethiazide 2.5 mg or 5 mg will be changed to indapamide (standard release) 2.5 mg.
2. Indapamide 2.5 mg will be changed to bendroflumethiazide 2.5 mg.
3. Indapamide 1.5 mg (modified release) will be changed to bendroflumethiazide 2.5 mg.

Practices assigned to indapamide will also be offered the choice of having any existing indapamide 1.5 mg modified release prescriptions switched to the more cost-effective 2.5 mg version, in keeping with the NICE guidelines.

The study pharmacist/technician will facilitate any prescription changes using established practice prescribing management procedures. All procedures for implementing switches are specified in a study Operations Manual. After the initial switching phase, all patients who newly require a thiazide-type diuretic for hypertension will be subject to the randomly assigned policy. Where possible, integrated electronic practice formularies will be updated to remind prescribers of the policy when issuing new prescriptions for thiazide/thiazide-like diuretics.

### Patient and public involvement

During the study pilot, feedback on study materials and methods was sought from patients and healthcare staff who contacted the study team. This feedback was incorporated into improved patient letters and switching methods. Our research unit’s public involvement group have discussed the research proposal and design, providing essential guidance on preferred patient communication methods. A study-specific patient involvement group will be formed to advise the study management group on ongoing patient communications and dissemination.

### Outcomes

Primary and secondary outcomes will be determined at an individual level and identified by individual-level record linkage of practice study populations to de-identified NHS datasets.

#### Primary outcome

MACE (major adverse cardiac event) is a widely accepted composite end-point employed for hypertension trials [27]. For this study, we define MACE as a hospitalisation for myocardial infarction, coronary revascularisation, stroke or heart failure, or vascular death.

#### Secondary outcomes

- Individual components of the primary outcome
- All-cause mortality
- Metabolic complications (hypokalaemia and hyponatraemia)
- New diabetes mellitus diagnoses

#### Tertiary outcomes

We will evaluate the method in terms of practice workload and acceptability, subsequent prescribing patterns, and treatment escalation.

### NHS data sets

These data will be provided by the electronic Data Research and Innovation Service (eDRIS), a part of Public Health Scotland. Permission to do this has been obtained from the Public Benefit and Privacy Panel for Health and Social Care (HSC-PBPP). If practices in other parts of the UK are recruited, this information will be obtained through the relevant national agencies.

Dispensed prescribing data for participating practices will be linked to Scottish hospitalisation (SMR01) and National Records of Scotland (NRS) death registration datasets. General practice data will complement these national datasets and allow more accurate ascertainment of secondary outcomes.

All analyses will use anonymised data within a secure research environment, accredited under the NHS Digital - Data Security and Protection Toolkit and the eDRIS National Safe Haven.

#### Clinical adjudication of cardiovascular events

The use of routinely collected NHS data for determining cardiovascular end-points for clinical trials is practical and cost-effective. It has been used extensively for large pragmatic trials with careful adjudication of all end-points by a specialised clinical committee [28–31]. However, as administrative data improves, it seems increasingly feasible to use the data without formal adjudication [32, 33]. We intend to adjudicate a proportion of outcome events identified from administrative data to estimate the sensitivity and specificity of administrative diagnostic coding in the study population. This process will require de-anonymisation of the individuals experiencing these events for clinical record retrieval. We have established and standardised procedures to do this based on previous and ongoing trials [34].

### Analyses and statistical methods

Using an intention-to-treat approach, the primary study analysis will use a baseline covariate-adjusted time-to-event individual patient-level analysis (proportional hazards model, if appropriate) to estimate the relative effectiveness of the two prescribing policies in preventing the primary outcome. We will adjust for a range of pre-specified covariates, including age, sex, co-morbidities and the Scottish Index of Multiple Deprivation [35]. This primary analysis will be of most use to policymakers in determining whether, as NICE guidance suggests, prescribers should be directed to favour one drug over the other.

We will perform a similar analysis using an as-treated approach to estimate the relative effectiveness of the two medications in preventing the primary outcome. This analysis will address the cited lack of direct comparative effectiveness evidence for these two drugs.

Randomisation and the large sample size (i.e. a large number of practices randomised) should reduce bias from an imbalance of baseline covariates. However, a range of sensitivity analyses will be performed to explore residual biases, including selection bias and differential adherence.

A detailed statistical analysis plan will be prepared and published.

#### Sample size

EVIDENCE is a cluster randomised study; statistical power, therefore, depends on the number of practices, the mean number of patients in each practice and the event rate and their variability between practices. We used data obtained from the Clinical Practice Research Datalink for the Bendroflumethiazide versus Indapamide for Primary Hypertension: Observational (BISON) study to estimate these variables [36]. First, we identified patients similar to the expected EVIDENCE study population and found an average of 200 individuals per practice, but with substantial variation in numbers between practices (standard deviation 140). We then defined an arbitrary index date and calculated the incidence of the EVIDENCE composite endpoint following that date. The overall event rate was 0.0305 per patient-year (0.0265 in patients with no previous outcome event and about six times higher in the 7% of follow-up time for patients with an earlier event). Finally, we estimated the between-practice standard deviation to be 0.0047.

Using these parameters, we ran simulations using random samples of 250 practices (sample size 50,000) with a mean follow-up time of 2 years and clinically plausible relative risks between treatments of 1.1 to 1.2. We also allowed the possibility that the BISON study overestimated the event rate by a factor of 2 or 3. We fitted mixed-effects models, in which variation between practices was treated as a random effect, and prior history was treated as a patient-level fixed effect, to estimate the confidence interval for the relative risk between treatments. Finally, we conducted a 2-sided test of the superiority of one treatment over the other. Assuming event rates are close to those found in the BISON study, a study size of 50,000 individuals (250 practices) will have 80% power to detect a relative risk of about 1.12 between treatments. If the event rate is 2 or 3 times lower, this study will have 80% power to detect relative risks of 1.16 and 1.19, respectively.

## Study conduct

### Study management group

An executive study committee will be constituted to guide the day to day running of the study. This group will consist of three of the principal investigators. An external advisor will be invited to join the study executive.

### Independent data monitoring committee

An independent data monitoring committee will be convened. The committee will comprise experienced pharmacoepidemiologists and clinicians with hypertension experience, as well as a trial statistician. The committee will receive unblinded data and will be expected to recommend changes to the conduct of the study, including early stopping of recruitment, based on their assessment of the relative risk/benefit of the study intervention. The committee will meet at least annually and will report to the study management group.

### Study management

A study pharmacist and clinical research fellow will oversee the study and will be accountable to the Chief Investigator (CI). The study pharmacist and clinical research fellow will be responsible for checking practice derived data for completeness, plausibility and consistency. However, this remains the overall responsibility of the CI. Any queries will be resolved by the CI or delegated member of the study team.

### Quality assurance

Since this study intervention is a randomised policy design, and there are no investigational medicinal products, no formal study monitoring is proposed. Principal investigators and institutions involved in the study will permit quality assurance audits or REC review as required by the sponsor. In the event of such a review, the investigators agree to allow the sponsor, representatives of the sponsor or regulatory authorities access to all records held by MEMO Research. Where such access is required, persons accessing study data will need to meet the standard conditions permitting such access. It should be noted that all person-specific study data will remain in GP practices and the eDRIS safe haven.

The EVIDENCE study will be undertaken by MEMO Research (www.memoresearch.com) and is sponsored by the University of Dundee and NHS Tayside (Sponsor R&D Number 2016CV12). The study has been approved by the East of Scotland Research Ethics Service (REC Number 17/ES/0016) and registered with ISRCTN (46635087). The Medicines and Health products Regulatory Agency has deemed that the EVIDENCE study is not a Clinical Trial of an Investigational Medicinal Product because it is an evaluation of prescribing policies for licensed medications.

### Protocol amendments

Any changes to the study protocol, except those necessary to remove an apparent, immediate hazard, will be reviewed and approved by the CI and sponsor. Amendments to the protocol will be submitted in writing for approval by the appropriate regulatory and ethical authorities before implementation.

### Dissemination

The results of this study will be disseminated through national and international conferences and papers. Authorship criteria will be based on recommendations of the International Committee of Medical Journal Editors. The results will also be shared with guideline committees, NHS organisations and patient groups.

## Discussion

EVIDENCE uses a new pragmatic trial method, combining essential elements of randomised clinical trials and observational analysis of routine clinical data collection and making use of routine NHS prescribing management activities. Cluster randomisation allows very large numbers of patients to be rapidly included in a study, facilitating an adequately powered study of short duration. This method provides a research infrastructure for rapid and highly efficient generation of evidence of comparative effectiveness of medications in situations where clinical equipoise exists.

In addition to the sample size considerations described above, cluster randomised trials are potentially vulnerable to significant imbalance in baseline covariates, leading to reduced statistical precision [24]. Although we have used block randomisation stratified by practice size to mitigate some cluster-level covariate imbalance, this may not be sufficient to account entirely for imbalance in cluster and individual patient-level baseline covariates, despite the relatively large number of clusters. For this reason, we plan a covariate-adjusted statistical analysis. Alternative methods of achieving balance at baseline, such as minimisation or covariate-constrained randomisation, should be considered for future trials using this design [24]. However, such approaches may be limited by lack of access to suitable data to calculate baseline covariate means and would increase the complexity of the study implementation in individual practices.

The primary intention-to-treat analysis will be susceptible to bias due to differential adherence. Although patients will not pay for either medication (all prescriptions are free within the NHS in Scotland), we may still expect differences in medication adherence between arms. For example, a patient experiencing minor symptoms may be more likely to attribute them to a new-to-them medication and discontinue or take the medication only intermittently, compared to a similar patient whose prescription has been unchanged. Therefore, the planned secondary as-treated analysis using dispensed prescribing data will be essential to interpreting the results.

Whilst we have tested the feasibility and acceptability of these methods, scaling the study nationally may be challenging. A successful national study will rely on close collaboration between the research team, participating practices, health boards and national data providers. This multi-disciplinary approach to health services research will be vital in achieving a genuinely learning healthcare system.

## Trial status

This manuscript describes approved protocol version 5, 26 August 2021. Recruitment to the pilot study began in October 2017, and full study recruitment is expected to continue until February 2024 (approximate).

## Data Availability

Not applicable (manuscript describes protocol only).

## Abbreviations

CPRD: Clinical Practice Research Datalink
EVIDENCE: Evaluating Diuretics in Normal Care
GDG: Guidelines development group
GP: General practitioner
NHS: National Health Service
NRS: National Records of Scotland
RCT: Randomised controlled trial
